# Detection of HHV-6 Virus in specimen of a ductal pancreatic adenocarcinoma with comparison in tumor and normal tissue

**DOI:** 10.1186/s13000-023-01402-z

**Published:** 2023-11-14

**Authors:** S. Warkentin, T. A. Braunschweig, D. Jonigk, I. Losen, M. A. Cassataro, M. Kleines

**Affiliations:** RWTH Aachen University DE, Aachen, Germany

## Abstract

**Aims:**

The association of human herpesvirus 6 (HHV-6) species with pancreatic cancer is controversially discussed. The aim of this study was to further investigate the postulated association and to identify the basis of HHV-6 DNA positivity reported for pancreatic cancer tissue.

**Methods:**

All samples of patients with pancreatic cancer (cancer and surrounding tissue) were analyzed for presence of HHV-6 DNA by PCR and then selected cases by immunohistochemistry.

**Results:**

Sixty eight per cent (68% = 52/77) of all patients were HHV-6 DNA positive in any of the samples, 49% (38/77) were positive in tumor tissue. Specimens of just one patient were HHV-6A DNA positive, all other patients were positive for HHV-6B. Immunohistochemical analysis of HHV-6 DNA positive samples did not reveal any specific HHV-6B protein positive tumor cell. In contrast, supposed immune cells presented intra- and peritumorally expressed HHV-6B-protein. The cause of presence of these cells in the tumor stroma is unknown, as of yet.

**Conclusions:**

HHV-6 DNA-positivity of pancreatic cancer tissue described by us and others is probably not due to the infection of pancreatic cells by HHV-6, but rather due to the migration of HHV-6 positive immune cells into the pancreas. Based on our data, we suppose that there is no direct evidence for HHV-6 as a causative agent of pancreatic cancer, but further in-depth studies (including investigation of immune status of patients) are necessary to make definitive conclusions.

## Introduction

Pancreatic adenocarcinoma is an aggressive epithelial tumor of the pancreas with an usually poor outcome. The etiology of this commonly late diagnosed cancer is not absolutely clear. Risk factors for the development of this cancer include: smoking [1], adiposity and diabetes [2]. Alcohol consumption as a risk factor is controversially discussed, but many researchers indicate it as an independent high-risk factor [3, 4]. Other published factors which may increase the risk of pancreatic cancer are contact with pesticides [5], herbicides, and fungicides [6]. Chlorinated hydrocarbons, chrome and its compounds [7], and other agents are discussed to influence the development of this disease.

Pancreatic cancer can be divided into sporadic (SPC) and familial (FPC) types. There are no distinct histological differences between carcinomas of these types. Individuals which are mutation carriers of the following genes have an increased lifetime risk of pancreas carcinoma (variable from 1 to 30%), beside other tumor entities within other syndromes: Adenomatous Polyposis Coli (APC), Ataxia-Telangiesctasia Mutated **(**ATM), BReast Cancer (BRCA) 1 and 2, Cyclin Dependent Kinase Inhibitor 2A (CDKN2A), MutL Homolog 1 (MLH1), MutS Homolog 2 (MSH2), MutS Homolog 6 (MSH6), PMS1 Homolog 2 (PMS2), Epithelial Cell Adhesion Molecule (EPCAM), Partner And Localizer of BRCA2 (PALB2), Serine/Threonine Kinase 11 (STK11), Tumor Protein p53 (TP53) [8]. There are some recommendations to keep these patients under surveillance to detect eventual tumors at an early stage [9]. Patients with hereditary pancreatitis have a high risk of pancreatic cancer several decades after first manifestation of pancreatitis [10]. Furthermore, von-Hippel-Lindau syndrome [11] and Fanconi anaemia [12] can predispose to pancreatic cancer.

Other factors of pancreatic cancer development, such as the role of infection with for example viral agents have been investigated. In a study of viral sequences in human cancer, researchers found one of the following members of the herpesvirus family in 13% of patients with pancreatic adenocarcinoma: Epstein-Barr Virus (EBV), Cytomegalovirus (CMV), and Human herpesvirus 6 (HHV-6), both in tumor and in normal tissue [13]. Another study, which used the bioinformatics system VirusScan, presented significantly higher abundances of these herpes virus species in tumor tissue of patients with gastrointestinal cancers in comparison to the adjacent normal tissue. The comparison of HHV6 in tumor and normal tissue of patients with pancreatic adenocarcinoma showed, in contrast, no virus abundance in either tissue types, after investigation of TCGA RNA-Seq data sets from CGHub, comprising 6.813 tumors and 559 adjacent normal samples across 23 cancer types [14]. A recent study regarding viral associations with human cancers which analyzed whole-genome sequencing (WGS) data of over 5.354 tumor–normal samples across 38 cancer types, and 1,057 tumor RNA-seq data across 25 cancer types identified 59 of 389 patients (15.2%) that were HHV-6A, HHV-6B and HHV-7-positive in their tumors (6% pancreas, 8% stomach, 8.3% colon/rectum tumors) and 90 of 389 patients (23.1%) that were positive in the non-malignant control samples. A correlation between virus detection and tumor development could not be confirmed [15].

HHV-6 viruses are members of the herpesvirus family and two species of HHV-6 have been defined: HHV-6A and HHV-6B. The latter one is known as the cause of Roseola infantum. Primary infection with HHV-6A has not been found to be associated with a distinct disease, so far. During infection with HHV-6A the infected cell is still able to produce cellular proteins. In contrast, HHV-6B blocks the synthesis of host cell protein completely, there is just synthesis of viral proteins. HHV-6 viruses, like all herpesviruses, persist after primary infection. Latency can be established in monocytes/macrophages and T-cells and reactivation can occur frequently [16]. HHV-6 viruses can integrate into the human genome. A large Japanese study showed that about 1% of the population (7.500 individuals investigated) have inherited virus (HHV-6B) in the form of a “cap” of chromosome 22q. The authors also discuss the reversibility of virus integration into the inherited human genome with the possibility of following production of infectious virus [17]. In another study, the prevalence of chromosomally integrated HHV6 genomes in the blood of UK blood donors was found to be 0.8% [18].

In this study we aimed to compare HHV-6-positivity in pancreatic tumor tissue and adjacent tissue and to identify the cause of HHV-6 positivity on the cellular level to allow a statement on the association between pancreatic cancer and HHV-6 infection.

## Materials and methods

### Preparation of samples

To investigate the tissue of patients with pancreatic adenocarcinoma 100 patients were identified in the archive of the Institute of Pathology of the University Hospital RWTH Aachen, which had been operated from 2010 to 2018 with the diagnosis of ductal adenocarcinoma of the pancreas. Immune status of the selected patients was unknown (see Table 1 with details of patient cohort). After approval of the study design by the local ethics committee of the University Hospital RWTH Aachen (EK 249/20) and privacy impact assessment (CTC-A Nr. 20–207) re-evaluation of hematoxylin and eosin (HE) slides of patients in regard to tumor and normal tissue was performed. As a next step, paraffin blocks were cut and areas with tumor and normal pancreatic tissue were separately macrodissected. Following DNA extraction (Maxwell RSC FFPE Plus DNA Kit) presence of HHV-6 DNA was investigated by PCR.

**Table 1:** Characteristics of cohort (numbers of patient of different gender (M: male, F: female), different tumor localization, grading and staging). Nota Bene 2017 Change of WHO T-Staging system depending on tumor size

Gender		Age distribution	Localization				Grading			Stage (NB 2017 WHO change)			
M: 51	F: 49	42—84	caput: 80	corpus: 1	cauda: 14	unclear/different areas: 5	no grading (after chemotherapy): 1	G2: 52	G3: 47	pT1: 2	pT2: 14	pT3: 81 (inlc. one case pT3m and one case ypT3)	pT4: 3

### Viral analysis

DNA extracts of 101 samples of tumor and normal tissue from selected patients were investigated for presence of HHV-6 DNA using the RealStar HHV-6 PCR Kit (Altona, Hamburg, Germany), following the manufacturer’s instructions. The primer was unknown.

### Immunohistochemical analysis

To verify the findings of the viral analysis via PCR, an immunohistochemical analysis of a selection of HHV-6 positive samples was performed using a mouse anti-human herpesvirus 6 B variant (HHV-6B) monoclonal antibody from Millipore, USA (catalog number: MAB8535, lot number: 3750154) as primary antibody. At first, we tested the antibody to choose the optimal dilution and pH. To exclude the unspecific background staining we used samples of tonsil and appendix. Afterwards we stained manually the samples, using our routine diagnostics protocol of immunhistochemical analysis (PH 6, dilution of primary antibody 1:400) with 3 µm thick slides and on slide controls of tonsil and appendix. The data sheet of the primary antibody did not contain the information about the localization of the staining. Other published articles presented the staining of HHV-6B primary antibody as a cytoplasmic reaction [19], also with Golgi distribution [20].

As a next step, we repeated the immunostaining with serial slides with HHV-6B and CD68 to verify the origin of positive stained cells. HHV-6B staining was performed manually as described above, and CD68 staining was performed in the DAKO Autostainer (with “ready to use” CD68 primary antibody, pH 6, from DAKO, IR613).

## Results

### Viral analysis

The analysis of 23/101 samples of patients with a pancreatic adenocarcinoma resulted in an inhibited HHV6-specific PCR and these samples were omitted from further investigation. The remaining samples (78/101) could be categorized in four groups regarding HHV-6 positivity in peritumoral and tumor tissue (Table 2). One patient was analyzed erroneously two times – with 2 different paraffin blocks (real remaining sample number is 77). Just one sample with HHV-6A DNA was identified (in tumor and normal tissue); all other virus-positive cases showed presence of HHV-6B DNA.

**Table 2:** HHV-6 DNA detection in different parts of specimens of patients with a pancreatic adenocarcinoma (*N* = number of samples)

	HHV-6 DNA-positive normal tissue	HHV-6 DNA-negative normal tissue
HHV-6 DNA-positive tumor tissue	*N* = 29 (38%)	*N* = 9 (12%)
HHV-6 DNA-negative tumor tissue	*N* = 14 (18%)	*N* = 25 (32%)

Interestingly, that the tissue of one same patient (after analysis of mistaken two different paraffin blocks of the same patient) showed different PCR results for HHV-6B – in on analysis both tissue types were negative, and in other – HHV-6B DNA was detected in the tumor tissue.

It was revealed that 68% (52/77) of all patients were HHV-6 DNA positive in any of the samples, 49% (38/77) were positive in tumor tissue. However, only for 12% (9/77) of the patients HHV-6 DNA positivity could be shown in tumor tissue exclusively. Characteristics of HHV-6 DNA positive patients are seen in the Table 3.

**Table 3:** Characteristics of HHV-6 DNA positive patients. Nota Bene 2017 Change of WHO T-Staging system depending on tumor size

Gender		Age distribution	Localization				Grading			Stage (NB 2017 WHO change)			
M: 26	F: 24	46—83	caput: 43	Corpus:1	cauda:5	unclear/different areas: 1	no grading (after chemotherapy): 1	G2:29	G3: 20	pT1: 2	pT2: 8	pT3: 38 (inlc. one case ypT3)	pT4: 2

### Immunohistochemical analysis

**Fig. 1:**
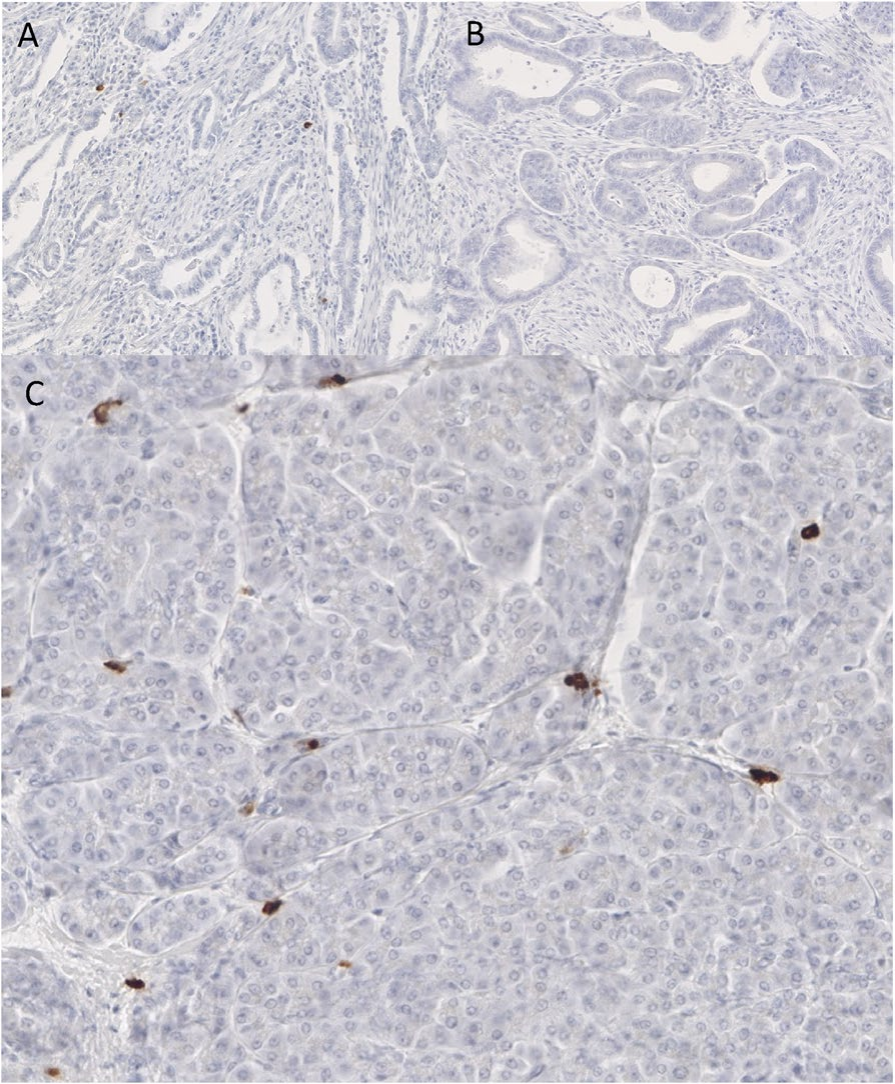
**A-C** HHV-6-DNA positive pancreas tissue investigated for HHV-6B protein by immunohistochemistry (brown are positive cells) (**A**: 66 × magnification – few positive immune cells in the tumoral stroma; **B**: 56 × magnification—no positive cells are visible intratumorally; **C**: 150 × magnification – normal pancreatic tissue with few HHV-6B protein positive stained immune cells)

An immunohistochemical analysis of randomly selected HHV-6 DNA positive samples (*N* = 4, DNA positivity in cancer and normal tissue) showed that tumor cells did not express HHV-6B protein. In contrast, supposed immune cells were positive for viral protein intratumoral and in the adjacent normal tissue. The staining showed a cytoplasmic pattern (Fig. 1A-C). Morphologically, due to a wide cytoplasm, we thought that these cells are rather macrophages than lymphocytes. Interestingly, just two of the four HHV-6 DNA positive samples investigated, also showed HHV6-B protein positivity intratumorally following immunohistochemical analysis. In contrast, the peritumoral normal tissue of all 4 samples showed a variable number of HHV-6B positive immune cells.

**Fig. 2:**
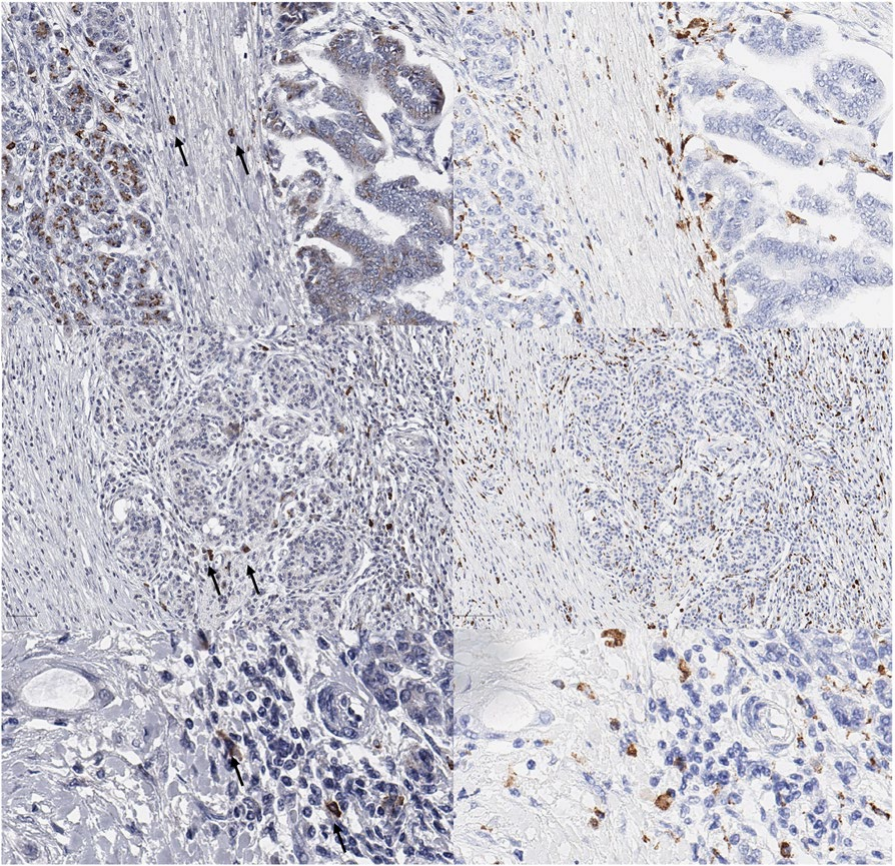
Three cases (upper, middle and low row) with comparison of HHV-6B (left) and CD68 (right) stained cells with no coexpression of cells (see arrows left)

To verify the origin of these cells, we repeated the analysis with serial section slides with immunostaining with HHV-6B and macrophage marker CD68. Unexpected, the repeated HHV-6B staining showed on the same tissue with initial negative stained tumor and normal tissue a cytoplasmic reaction (rather unspecific) in normal and cancer tissue beside initial seen positive “immune cells” by staining in DAKO Autostainer and also repeated by manual staining (Fig. 2). The comparison of serial section slides indicates that HHV-6B-positive cells do not express CD68, so they are most probably not macrophages, but probably other immune (?) cells.

## Discussion

Pancreatic ductal carcinoma is a highly aggressive tumor, which is usually diagnosed, due to anatomic features, in the late stage and has a poor prognosis. Etiologically different agents, including infections, are discussed in the literature.

It is well known, that the members of herpesvirus-family can persist in the human body following primary infection [21] in the form of episomes. HHV-6 can integrate into the telomeric region of chromosomes [22]. Some patients, especially those receiving immune suppression, can also develop herpesvirus-associated malignancies (such as Kaposi sarcoma with HHV-8 and nasopharyngeal carcinoma with Epstein-Barr virus via induction of lymphangiogenesis [23]).

There is a wide spectrum of studies, which have reported on the role of HHV-6 in malignancies, such as Hodgkin and Non-Hodgkin lymphoma, leukemia, neurological, gastroenterological, gynecological, head and neck cancers [24]. However, the number of studies which analyzed HHV-6 association with/presence in pancreatic cancer is small. One study detected herpes virus members, including HHV-6, both in cancer and in normal tissue in 13% of cases [12]. In contrast, no HHV-6 virus abundance in both tissue types, in a study of TCGA RNA-Seq data sets, comprising > 6.000 tumors and > 500 adjacent normal samples across approximately 20 cancer types, was found [13]. A further study based on whole genome sequencing analysis found 6% of analyzed patients with pancreatic cancer being HHV-6A, HHV-6B or HHV-7-positive. Therefrom approximately 15% were positive in the tumors and approximately 23% in the non-tumorous tissue. At the same time, no correlation between virus detection and tumor development could be confirmed [14].

Based on these findings, we wanted to analyze, whether any commonality of HHV-6-virus and pancreatic cancer could be found.

We performed a HHV-6 specific viral analysis of 101 samples of patients with pancreatic ductal carcinoma in tumor and in surrounding normal pancreatic tissue. Results of our analysis with detection of HHV-6 DNA in tumor and surrounding tissue are consistent with other analyses published.

So far, all studies which analyzed HHV-6 in pancreatic cancer, relied on virus-specific PCR. An identification of the individual HHV-6 positive cells was not performed. The tumor tissue is made up by a tumor microenvironment with different components: cancer cells, incl. stem cells, cancer-associated fibroblasts, pancreatic stellate cells, different immune cells, and extracellular matrix [25]. Thus, we performed a verifying immunohistochemical analysis of selected positive cases to pinpoint the cells of interest and found that the tumor cells did not express specifically HHV-6B; however, intra- and peritumorally visible immune (?) cells were consistently HHV-6 antigen positive. We supposed initially due to morphology of these cells that they are rather macrophages, then lymphocytes. An additional analysis with serial sections showed no coexpression of HHV-6B with a macrophage marker CD68. So, further investigation with serial sections or multiple staining of HHV-6B and other immune cells markers are necessary to better understand the origin of these cells.

Also, it is important to verify the staining in the tumor and normal pancreatic tissue (in our investigation rather unspecific) with other primary antibody and a large number of investigated cases.

Our findings indicate at first sight that no direct association between HHV-6 infection and pancreatic cancer can be proposed.

On the other hand, there are virus-induced neoplasias, such as an angioimmunoblastic T cell lymphoma (AITL), where malignant T cells are negative for EBV but surrounding B immunoblasts demonstrate active EBV-infection [26]. Therefore, at this point and considering to limitation factors such as a small patient cohort, further investigations are necessary to evaluate the tumor milieu of pancreatic cancer and make definitive conclusions.

The HHV-6 PCR positive cases can be explained by migrating HHV-6 positive immune cells, which are present in the tumor microenvironment and the surrounding tissue. It is known that HHV-6 can productively infect different immune cell types including CD4 + T lymphocytes, CD8 + T cells, gammadelta T cells, and natural killer cells. Other immune cells like macrophages and dendritic cells can also be infected by HHV-6, but typically in a nonproductively manner [27].

The identification of positive cells via specific antibodies raised for the detection of HHV-6B protein in HHV-6A DNA positive specimens can be explained by the cross-reaction of the primary antibody, which can also probably detect the HHV-6A protein, besides the HHV-6B protein.

In summary, in this study, we correlated findings of PCR and immunohistochemistry for HHV-6 in the tumor and peritumoral normal tissue of patients with pancreatic cancer, in order to show or to exclude an association between this virus and pancreatic cancer. We found HHV-6A and -6B PCR products in cancer tissue, but rather no specific expression of HHV-6B protein on cells of the ductal carcinomas themselves. The HHV-6B protein was expressed in probably immune cells (not macrophages), which could migrate into tumor tissue. Therefore, we postulate that there is no direct correlation between HHV-6B virus infection and pancreatic cancer, based on our findings. However, in view of some neoplasms where neoplastic cells do not harbor the virus and surrounding cells are infected further studies of tumor microenvironment with knowledge of immune status of patients are necessary.
